# Manual of Hygiene

**Published:** 1904-12

**Authors:** 


**Manual of Hygiene.** By W. H. Hamer, M.D., D.P.H. (Cantab.). Pp. xii., 622. London: J. & A. Churchill. 1902.

We must congratulate Dr. Hamer upon the masterly way in which he has dealt with his subject, and upon the general arrangement and style of his book, which combines the compactness of a text-book with the lucidity and fluency of a monograph. He has wisely refrained from taxing his reader with too much technical detail, especially where such subjects as bacteriology and chemistry are concerned, but on the other hand he might well have devoted more space to the discussion of such important questions as the position of Gärtner's bacillus in reference to Widal's reaction, or of the $D^2$ bacillus in connection with epidemics of diphtheria.

Many of the chapters might be fuller. The chapter on Air, for example, contains eighty-seven pages, and of these barely four are consigned to the important topic of vitiating dusts and vapours inseparable from many of our manufacturing industries. No mention, for instance, is made of the dyspepsia common among potters, or of the skin ulcers found in those engaged in the printing process known as "bronzing," &c. And more stress might have been laid upon the fact that factories, owing to their dusts and fumes, should be much more liberally lighted and ventilated than ordinary dwelling-houses.

The chapter on Dwellings, Schools and Hospitals is undoubtedly excellent.

We should have welcomed a more exhaustive discussion upon the relative merits of the various methods of sewage disposal, which subject has been dismissed in nine pages! A full and detailed account of the biological process, with diagrams of tanks and plant, would have been interesting.

Infectious diseases are conveniently arranged in alphabetical order, but here again some omissions are noticeable. No mention is made of epidemic colitis, so common in large institutions such as Unions; and in reference to sewer gas and its bearing on enteric fever, the ubiquitous fly is not mentioned as a probable conveyor of infection.

The section which deals with statistics has been rendered not only readable but entertaining, which fact alone speaks volumes for Dr. Hamer's capabilities as an author.

The book is, we feel confident, certain to take a high place as a standard work.

